# Supplementary material for: Optimization of Copper Stain Removal from Marble through the Formation of Cu(II) Complexes in Agar Gels
Source: Gels. 2021 Aug 6;7(3):111. doi: 10.3390/gels7030111 (PMC8395995; doi:10.3390/gels7030111)
Supplement: Supplementary file 1 [file gels-07-00111-s001.zip › gels-1258253-supplementary.pdf]

Supplementary Materials

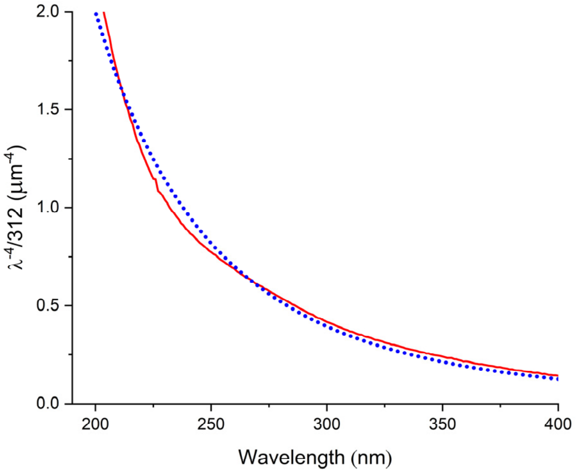

Figure S1 - UV-Vis absorbance spectrum of pure agar gel (dotted curve), compared with a curve representing the λ^{-4} dependence of the intensity of scattered light.

The pure agar gel is pale gray, a colour which can suggest light scattering. In order to check this hypothesis, in Figure S1 the spectrum of the pure agar gel is compared with a λ^{-4} law, commonly used for describing Rayleigh scattering. The two curves nicely match, suggesting light scattering as indeed responsible for the observed spectral lineshape**.**

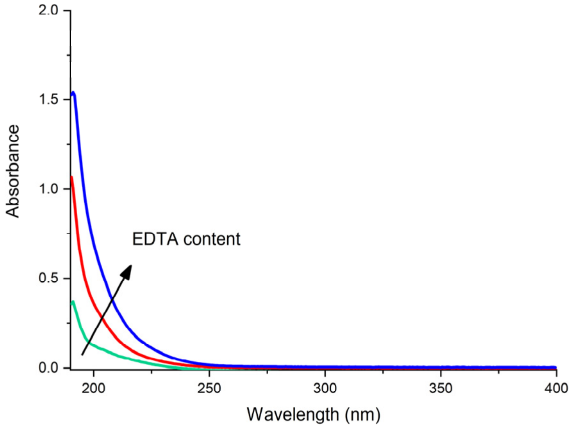

Figure S2 - UV-Vis absorbance spectra of free aqueous solutions containing EDTA at increasing concentrations (5 x 10^{-4}, 1.5 x 10^{-3}, 2 x 10^{-3} M, corresponding to 0.015, 0.044, 0.058 % w/w, respectively). The optical path in the cuvette is 1 cm.

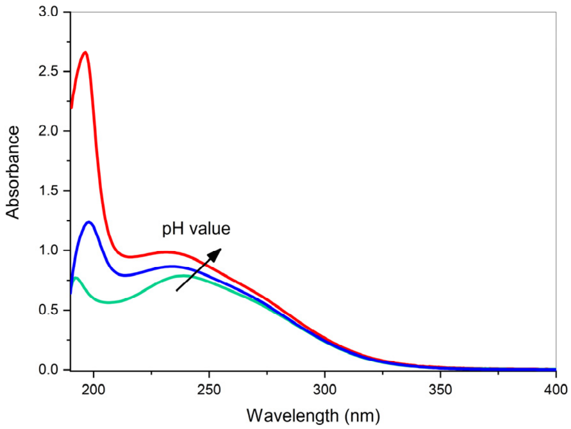

Figure S3 - UV-Vis absorbance spectra of free aqueous solutions containing both $CuSO_4$ and EDTA in molar ratio 1:1 at increasing pH (5.0, 6.0 and 8.0). The optical path in the cuvette is 1 cm.

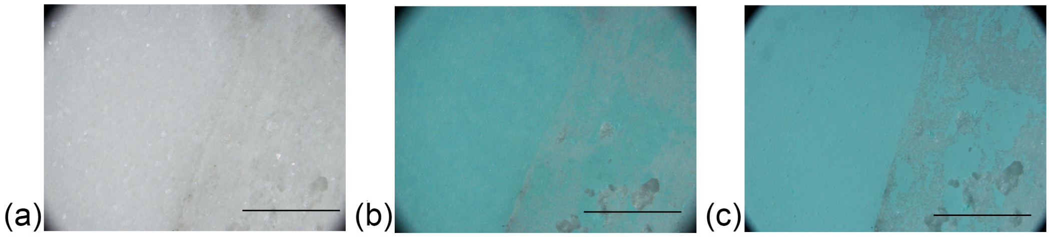

Figure S4 - Microscopic image of a marble specimen (a) before staining, (b) after staining with brochantite and (c) after treatment with agar gel-EDTA for 60 minutes (magnification 10×; scale bar 5 mm). The cleaning action could not be considered complete.

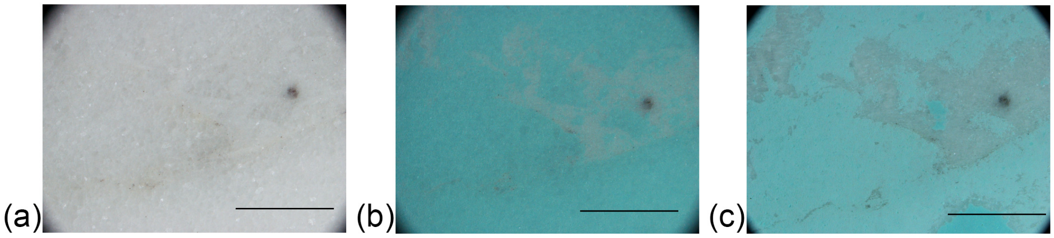

Figure S5 - Microscopic image of a marble specimen (a) before staining, (b) after staining with brochantite and (c) after treatment with agar gel-TAC for 60 minutes (magnification 10×; scale bar 5 mm). The cleaning action could not be considered complete.

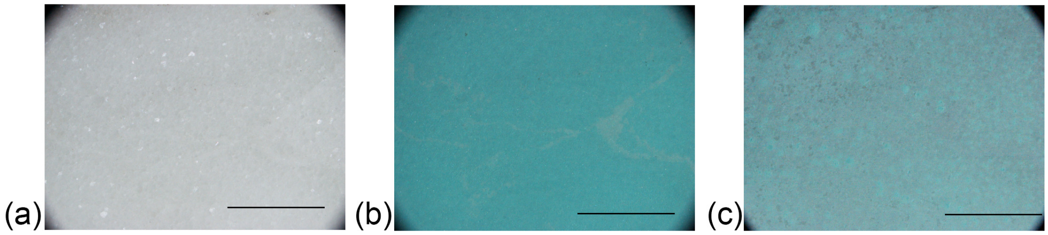

Figure S6 - Microscopic image of a marble specimen (a) before staining, (b) after staining with brochantite and (c) after treatment with agar gel-ALA for 60 minutes (magnification 10×; scale bar 5 mm). The cleaning action could not be considered complete.

Hydroxide solubility and complexation

The general framework for the interpretation of the data include the simultaneous equilibria involving copper complexation by the ligands and its precipitation as an hydroxide, mainly $Cu(OH)_2$. The complexation and precipitation reactions do compete in solution, and both are favored when the pH is raised because of the deprotonation of the ligand and the higher hydroxyl ion concentration, respectively. As an example, when EDTA is used as the ligand, the following system of equations holds under the used experimental conditions:

$$K'_{f\ CuEDTA}=\frac{[CuEDTA]}{[Cu^{2+}][EDTA]}$$

$$K_{PS\ Cu(OH)_2}=[Cu^{2+}][OH]^2$$

$$[EDTA]= 5 \times 10^{-4}\ M$$

$$C_{Cu}=[Cu^{2+}]+[CuEDTA]+[Cu(OH)_2]= 5 \times 10^{-4}\ M$$

Where K'_f is the conditional stability constant calculated for the investigated pH, i.e. the K_f times the side reaction coefficient α_{EDTA4-}. Simulation at pH 10.0 (the highest value employed in this study) by the equilibrium software Visual MINTEQ (version 3.1) indicate the complete complexation of copper by EDTA (99% of copper is in the form $CuEDTA^{2-}$ and 1% in the form $CuOHEDTA^{3-}$) and undersaturation in copper hydroxide. This result is in accordance with the experimental evidence indicating no precipitation of the hydroxide and complexation by EDTA (see text).
